# Mitochondrial dysfunction in an animal model of diabetic neuropathy is associated with a reduction of neurosteroid synthesis.

**DOI:** 10.12688/f1000research.11056.2

**Published:** 2018-03-09

**Authors:** Stephen R. Humble

**Affiliations:** 1 Department of Anaesthetics and Pain Management, Charing Cross Hospital, Imperial College NHS Healthcare Trust London, London, W6 8RF, UK; 2 Imperial College London, Charing Cross Campus, Margravine Road, London, W6 8RP, UK

**Keywords:** Diabetes, neuropathic, pain, neurosteroid, benzodiazepine, ob/ob, mitochondria, TSPO, GABA

## Abstract

**Background** *:* Recent work in a model of diabetic neuropathy revealed that layer 2/3 cortical pyramidal neurones of the pain pathway exhibited reduced endogenous neurosteroid modulation of the GABA _A_R and exogenously applied neurosteroids had an exaggerated impact. It is postulated that this is related to reduced precursor synthesis, due to mitochondrial dysfunction in diabetic neuropathy. Benzodiazepines are also known to activate neurosteroidogenesis by binding to mitochondrial translocator protein (TSPO). This study explored the differential effect of diazepam on GABA _A_R modulation via neurosteroidogenesis in diabetic and wild type (WT) mice.

**Methods** *:* Whole-cell patch-clamp technique was used on slices of neural tissue. Electrophysiological recordings were obtained from layer 2/3 cortical pyramidal neurons of the pain pathway from mice with type-II diabetic neuropathy ( *ob/ob*) and WT controls aged 60-80 days.

**Results** *:* There was a key difference in the response of the WT and *ob/ob* cortical neurons to simultaneous incubation with diazepam and flumazenil. In contrast, diazepam and the 5a-reductase inhibitor finasteride, individually or in combination, produced the same response in both strains.

**Conclusions** *: *The exaggerated effect of diazepam on GABAergic inhibitory tone in the *ob/ob*, despite the presence of the GABA _A_R benzodiazepine antagonist flumazenil is likely observed due to physiological upregulation of key neurosteroidogenic enzymes in response to the reduced pregnenolone synthesis by the mitochondria. By increasing pregnenolone via TSPO activation, it is possible to promote enhanced neurosteroidogenesis and increase GABAergic inhibitory tone via an alternate route. In diabetic neuropathy, mitochondrial dysfunction may play an important role. Enhancing the GABAergic neurosteroid tone could be of potential therapeutic benefit.

## Introduction

Diabetic neuropathy is a common cause of painful neuropathy, and treatment is often suboptimal because the underlying aetiology is poorly understood. Peripheral and central sensitisation are implicated in the development of neuropathic pain with neuroplasticity occurring at multiple levels of the pain pathway ( Harvey & Dickenson, 2008). GABAergic neurones at all levels of the pain pathway have a vital role in the transmission of painful stimuli in the perception of pain itself ( D’Mello & Dickenson, 2008). Endogenous and exogenous neurosteroids may act as potent positive allosteric modulators of GABA _A_ receptors (GABA _A_Rs) and consequently exhibit analgesic, anxiolytic, anticonvulsant, and sedative properties ( D'Hulst *et al.*, 2009).

**Figure 1.**
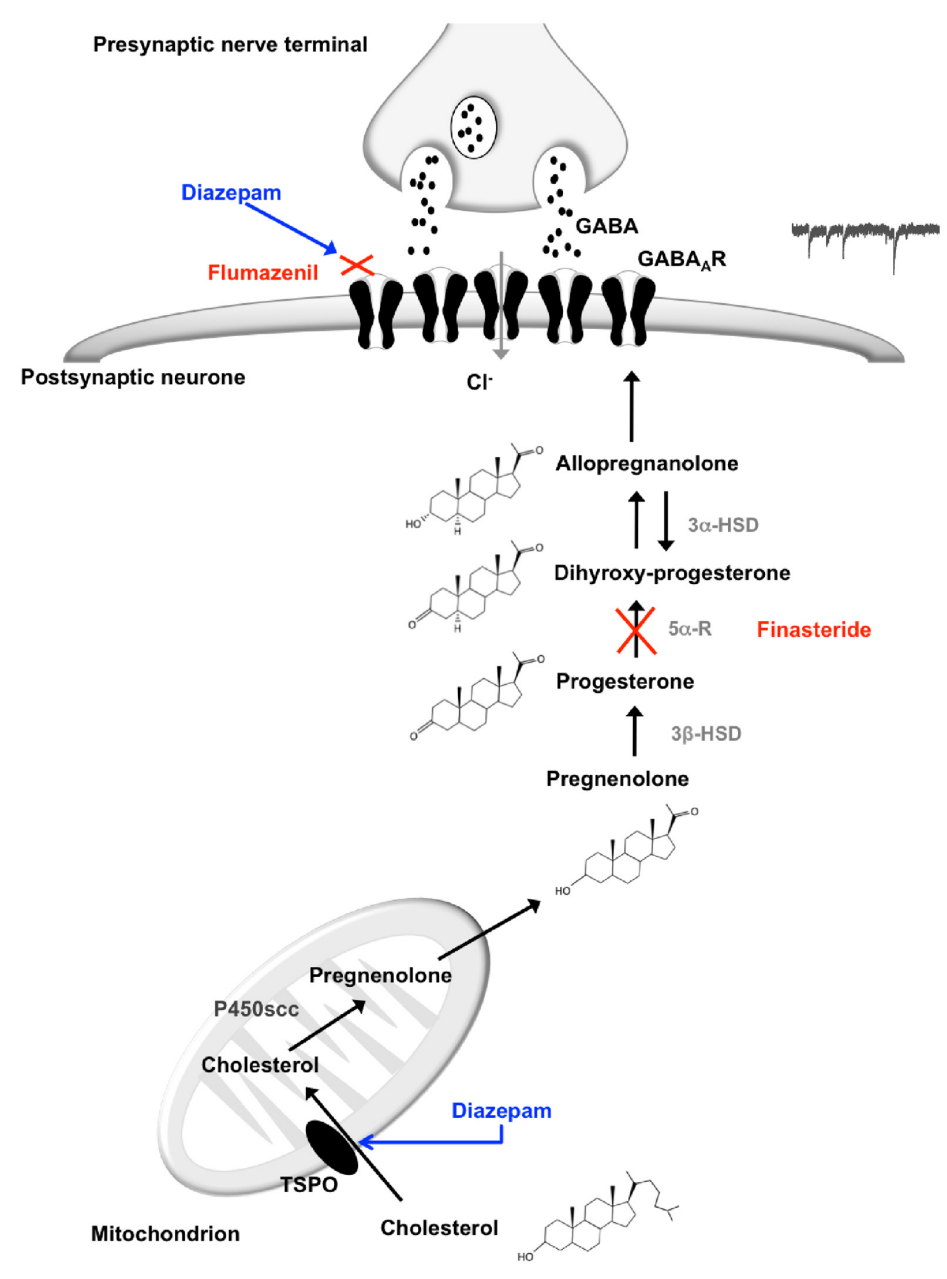
Modulation of the GABA _A_R by endogenous neurosteroids. Cholesterol is taken through the mitochondrial membrane by the translocator protein (TSPO) where it is converted to pregnenolone by the cytochrome P450 side chain cleavage enzyme. Pregnenolone is converted to progesterone by 3β-hydroxysteroid dehydrogenase (3β-HSD), which is in turn reduced to dihydroxyprogesterone by 5α-reductase (5α-R). Dihydroxyprogesterone is converted to allopregnanolone by 3α-hydroxysteroid dehydrogenase (3α-HSD). Postsynaptic GABA _A_Rs are activated by GABA that has been released from vesicles in the presynaptic nerve terminal. GABA induces a conformational change of the GABA _A_R, opening its central channel and thereby allowing the passage of chloride ions and the subsequent generation of miniature inhibitory postsynaptic currents (mIPSCs). The negative chloride ions induce hyperpolarisation of the neuronal membrane, which mediates neuronal inhibition. Neurosteroids, such as the active compound allopregnanolone, modulate GABA _A_R function and facilitate inhibition of the neuronal membrane. ( Humble, 2013). The translocation of cholesterol into the mitochondria by TSPO is the first rate-limiting step and is enhanced by the presence of specific ligands such as diazepam. Thus diazepam may enhance GABA _A_R modulation by binding to the GABA _A_R directly and separately by increased neurosteroidogenesis. This modulation may be selectively inhibited at the GABA _A_R itself by the antagonist flumazenil and separately by the 5α-R inhibitor finasteride, which inhibits neurosteroidogenisis.

Within inhibitory synapses, the presynaptic fusion of a single vesicle releases the inhibitory neurotransmitter GABA to activate synaptic GABA _A_Rs. Under voltage-clamp conditions this causes a miniature inhibitory postsynaptic current (mIPSC). Drugs that enhance GABA _A_R function cause a prolongation of the mIPSC decay phase. Recent work in a model of type-II diabetic neuropathy ( *ob/ob*) revealed that layer 2/3 cortical pyramidal neurones of the pain pathway exhibited reduced endogenous neurosteroid modulation of the GABA _A_R, and exogenously applied neurosteroids had an exaggerated impact ( Humble, 2016a). The mechanism responsible appeared unrelated to GABA _A_R sensitivity, but instead was associated with a reduction of neurosteroid precursors, such as pregnenolone, which is metabolised sequentially to the active compound allopregnanolone ( Figure 1) ( Humble, 2013; Humble, 2016a). Pregnenolone is synthesised in the mitochondrion from its precursor cholesterol by the side chain cleavage enzyme P450 located in the inner mitochondrial membrane ( Do Rego *et al.*, 2009; Schumacher *et al.*, 2012), and it is postulated that diabetic neuropathy may be associated with a reduction in mitochondrial activity. Cholesterol is translocated across the mitochondrial membrane by the 18 kDa translocator protein (TSPO) in a coordinated fashion with the steroidogenic acute regulatory (StAR) protein ( Do Rego *et al.*, 2009; Gatliff & Campanella, 2016; Rupprecht *et al.*, 2010; Stocco *et al.*, 2017; Figure 1).

The present study explored the impact of the benzodiazepine diazepam, a positive allosteric modulator of the GABA _A_R ( D’Hulst *et al.*, 2009), on GABA _A_R modulation via neurosteroidogenesis in diabetic and wild type (WT) mice. Benzodiazepines are also known to activate neurosteroidogenesis by binding to TSPO ( Rupprecht *et al.*, 2010; Tokuda *et al.*, 2010).

## Methods

The methods are identical to those published by the same author previously ( Humble, 2016a), with the exception of the drugs diazepam and flumazenil, which were not used in the previous study. Diazepam and flumazenil were purchased (Tocris, Bristol UK) and prepared as concentrated stock solutions in dimethyl sulfoxide before being added to the artificial extracellular solution as per the previous study ( Humble, 2016a).

## Results

### Prolonged exposure (2 hrs) of mature cortical neurones to diazepam (1μM) in the presence of flumazenil had an exaggerated effect on the cortical GABA _A_R-mediated mIPSCs of *ob/ob* mice in comparison to WT mice

**Figure 2.**
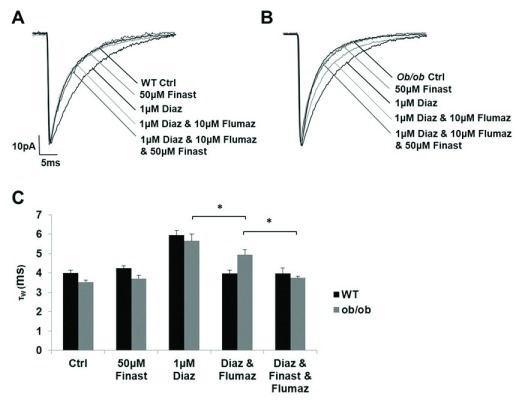
Prolonged exposure (2 hrs) of mature cortical neurones to diazepam (1 μM) in the presence of flumazenil had an exaggerated effect on the cortical GABA _A_R-mediated mIPSCs of *ob/ob* mice in comparison to WT mice. ( **A**) Superimposed exemplar averaged GABA _A_R-mediated miniature inhibitory postsynaptic currents (mIPSCs) acquired from a representative WT cortical neurone and from equivalent neurones after ~2 hours pre-incubation of the brain slice with diazepam (1 μM), flumazenil (10 μM) and finasteride (50 μM). ( **B**) Superimposed exemplar averaged GABA _A_R-mediated mIPSCs acquired from a representative *ob/ob* cortical neurone and from equivalent neurones after ~2 hours pre-incubation of the brain slice with diazepam (1μM), flumazenil (10 μM) and finasteride (50 μM). ( **C**) Histogram illustrating that flumazenil is able to prevent the effect of diazepam to prolong the duration of the GABA _A_R-mediated mIPSC in WT cortical neurones, but only has a partial efficacy in *ob/ob* cortical neurones (τ _w_ in ms; one-way ANOVA, *P* >0.05; *Post hoc* Newman Keul’s test). The persisting effect of diazepam in the presence of flumazenil in the *ob/ob* mice could be prevented by the presence of the 5α-reductase enzyme inhibitor finasteride (τ _w_ in ms; one-way ANOVA, *P* >0.05; *Post hoc* Newman Keul’s test). Ctrl = control; Finast = finasteride; Diaz = diazepam; Flumaz = flumazenil.

Whole-cell voltage-clamp recordings were made in L2/3 cortical neurones of WT and *ob/ob* mice after at least two hours of incubation with diazepam, flumazenil and finasteride. Diazepam alone had the same effect on both strains of mice. In the WT mice, flumazenil inhibited the effect of diazepam (τ _W_: control = 4.0 ± 0.1 ms, n = 35; finasteride 50 μM = 4.2 ± 0.1 ms, n = 7; diazepam 1 μM = 5.9 ± 0.2 ms, n = 6; flumazenil 10 μM & diazepam 1 μM = 4.0 ± 0.2 ms, n = 7; flumazenil 10 μM, finasteride 50 μM & diazepam 1 μM = 4.0 ± 0.3 ms, n = 6; One-way ANOVA, *P* <0.05; *post hoc* Newman Keul’s test revealed a difference only for diazepam 1 μM, *P* <0.05; Figure 2). By contrast, in the *ob/ob* mice, flumazenil only partially inhibited the effect of diazepam, and the persisting effect of diazepam in the presence of flumazenil in the *ob/ob* mice could be prevented by the presence of the 5α-reductase enzyme inhibitor finasteride (τ _W_: *ob/ob* control = 3.5 ± 0.1 ms, n = 25; finasteride 50 μM = 3.7 ± 0.2 ms, n = 6; diazepam 1 μM = 5.7 ± 0.3 ms, n = 6; flumazenil 10 μM & diazepam 1 μM = 4.9 ± 0.3 ms, n = 6; flumazenil 10 μM, finasteride 50 μM & diazepam 1 μM = 3.7 ± 0.1 ms, n = 5; One-way ANOVA, *P* <0.05; *post hoc* Newman Keul’s test revealed significant intergroup differences for the flumazenil groups, *P* <0.05; Figure 2).

## Discussion

Layer 2/3 cortical neurones from mature type-II diabetic *ob/ob* are known to have a reduced endogenous pregnane-derived neurosteroid tone in comparison to strain matched WT controls ( Humble, 2016a). The present data indicate that by promoting the uptake of pregnenolone’s precursor cholesterol by the mitochondria, via TSPO, diazepam may rescue the reduced neurosteroid tone. The restored neurosteroid tone could re-establish GABA _A_R-mediated neuro-inhibitory tone in cases of neuropathic hypersensitivity. With specific reference to these data, the key result is the difference in response of the WT and *ob/ob* to simultaneous incubation with diazepam and flumazenil. In contrast, diazepam and the 5α-reductase inhibitor finasteride individually or in combination produced the same response in both WT and *ob/ob*. This may be interpreted as follows: in the WT, the primary effect of diazepam incubation is direct allosteric modulation of the GABA _A_R, with negligible contribution from neurosteroidogenesis via mitochondrial TSPO activation. In comparison, diazepam has an exaggerated effect on GABAergic inhibitory tone in the *ob/ob*, despite the presence of the GABA _A_R benzodiazepine antagonist flumazenil. This effect is likely observed due to physiological upregulation of the key rate-limiting enzymes involved in neurosteroidogenesis in response to the reduced pregnenolone synthesis by the mitochondria ( Figure 1; Humble, 2016a). Thus by increasing the availability of the neurosteroid precursor pregnenolone via TSPO activation, it is possible to promote enhanced neurosteroidogenesis and thereby increase GABAergic inhibitory tone via an alternate route. Benzodiazepines modulate the GABA _A_R by binding to the α-γ subunit interface ( D'Hulst *et al.*, 2009), while neurosteroids bind the GABA _A_R from a cavity within the α-subunit domain and modulate it directly via the α-β subunit interface ( Hosie *et al.*, 2006). There have already been a number of other studies of ligands for the mitochondrial TSPO, as this is a promising target ( Gatliff & Campanella, 2016; Giatti *et al.*, 2009; Papadopoulos & Lecanu, 2009; Rupprecht *et al.*, 2010; Zhang *et al.*, 2016). Considering the findings in this paper alongside previous work ( Humble, 2016a) it appears that mitochondrial dysfunction may play an important role in the development of type 2 diabetic neuropathy. In this context, it follows that enhancing the GABAergic neurosteroid tone directly or indirectly could be of potential therapeutic benefit for diabetic neuropathic pain and hypersensitivity.

## Data availability

The data referenced by this article are under copyright with the following copyright statement: Copyright: © 2018 Humble SR

Data associated with the article are available under the terms of the Creative Commons Zero "No rights reserved" data waiver (CC0 1.0 Public domain dedication). 

Open Science Framework: Dataset of ‘Neurosteroids are reduced in diabetic neuropathy and may be associated with the development of neuropathic pain’, doi: 10.17605/osf.io/bk3tw ( Humble, 2016b). Raw data for the present study can be found in Diazepam.zip.

Please refer to ( Humble, 2016a) for details of standard software used for data analysis.

## Supplementary material

Supplementary Tables 1 and 2: Analysed raw data.
