# Supplementary material for: Mitochondrial dysfunction in an animal model of diabetic neuropathy is associated with a reduction of neurosteroid synthesis
Source: F1000Res. 2018 Mar 9;6:506. Originally published 2017 Apr 18. [Version 2] doi: 10.12688/f1000research.11056.2 (PMC5861509; doi:10.12688/f1000research.11056.2)
Supplement: Supplementary file 1 [file f1000research-6-15396-s0000.tgz › 0b619211-f27f-4d30-a31a-8584abdcfd0c.zip!/Supplementary Table 1.doc]

Table 1. Flumazenil (Flumaz) prevents the effect of diazepam (Diaz) incubation treatment on GABA_A_R mIPSCs of L2/3 cortical neurones (P60-75) of WT mice, but only has a partial effect on neurones of *ob/ob* and *db/db* mice

|  | **WT**  **Control**  **( n=35)** | ***Ob/ob***  **(n=25)** | **WT**  **1μM**  **Diaz**  **( n=6)** | ***Ob/ob***  **1μM**  **Diaz**  **( n=6)** | **WT**  **1μM Diaz**  **& 10μM Flumaz**  **(n=7)** | ***Ob/ob***  **1μM Diaz**  **& 10μM Flumaz**  **(n=6)** | **WT**  **1μM Diaz**  **& 10μM Flumaz**  **& 50μM Finast**  **(n=6)** | ***Ob/ob***  **1μM Diaz**  **& 10μM Flumaz**  **& 50μM Finast**  **(n=5)** |
| --- | --- | --- | --- | --- | --- | --- | --- | --- |
| **Peak amp (pA)** | -59 ± 2 | -66 ± 2 | -66 ± 2 | -65 ± 3 | -56 ± 3 | -70 ± 3 | -56 ± 3 | -55 ± 2 |
| **Rise Time (ms)** | 0.4 ± 0.1 | 0.4 ± 0.1 | 0.5 ± 0.1 | 0.5 ± 0.1 | 0.4 ± 0.1 | 0.4 ± 0.1 | 0.4 ± 0-1 | 0.4 ± 0.1 |
| **Charge transfer (fC)** | -242 ± 9 | -255 ± 12 | -405 ± 17 | -376 ± 25 | -236 ± 16 | -352 ± 18 | -232 ± 21 | -223 ± 12 |
| **T70 (ms)** | **5.2 ± 0.2** | **4.7 ± 0.1** | ****8.1 ± 0.3** | ****7.8 ± 0.5** | **5.5 ± 0.2** | ****6.6 ± 0.4** | **5.4 ± 0.3** | **5.2 ± 0.2** |
| **Tau w (ms)** | **4.0 ± 0.1** | **3.5 ± 0.1** | ****6.0 ± 0.2** | ****5.7 ± 0.3** | **4.0 ± 0.2** | ****4.9 ± 0.3** | **4.0 ± 0.3** | **3.7 ± 0.1** |
| **Freq (Hz)** | 17 ± 2 | 21 ± 3 | 18 ± 2 | 17 ± 2 | 16 ± 2 | 18 ± 2 | 16 ± 3 | 15 ± 2 |

***P* < 0.05; One-way ANOVA (with *Post hoc* Newman Keul’s test) in comparison to strain-matched control (P60-75)
