# Supplementary material for: Mitochondrial dysfunction in an animal model of diabetic neuropathy is associated with a reduction of neurosteroid synthesis
Source: F1000Res. 2018 Mar 9;6:506. Originally published 2017 Apr 18. [Version 2] doi: 10.12688/f1000research.11056.2 (PMC5861509; doi:10.12688/f1000research.11056.2)
Supplement: Peer review history

# Peer review: Referee response for version 2

**DOI:** 10.5256/f1000research.15396.r31739

**Published:** 2018-03-20

**Authors:** Slobodan M. Todorovic

**Affiliations:** 1 Department of Anesthesiology, School of Medicine, University of Colorado Anschutz Medical Campus, Aurora, CO, USA

I have read this submission. I believe that I have an appropriate level of expertise to confirm that it is of an acceptable scientific standard.

# Peer review: Referee response for version 1

**DOI:** 10.5256/f1000research.11924.r23003

**Published:** 2017-06-06

**Authors:** Pascal Darbon

**Affiliations:** 1 Institut des Neurosciences Cellulaires et Intégratives (INCI), UPR 3212 CNRS, Centre National de la Recherche Scientifique and University of Strasbourg, Strasbourg Cedex, France

The present article is a follow-up of Humble’s previous paper in F1000Research ( *Humble* SR. Neurosteroids are reduced in diabetic neuropathy and may be associated with the development of neuropathic pain [version 1; referees: 1 approved, 2 approved with reservations]. *F1000Research* 2016, **5**:1923 doi: 10.12688/f1000research.9034.1). The current article could be considered as an extended version providing further clarification on involved mechanisms. Therefore to fully understand the presented results, I recommend reading first the previous article.

 However, in order to reach a broader leadership illustration and description could be improved. Figure 1 could be improved by highlighting the difference with Humble’s 2016 paper; action sites of Diazepam, Flumazenil and Finasterid could be indicated. Likewise, results description focuses on difference between both mice strains without fully presenting individual results (Finast, Diaz, Flumaz…). This required a strong background in the field.

 My last comment concerns the primary effect of diazepam on GABA _A_R. The experiment consists in a 2 h incubation and it has been shown ( *in vivo* in other brain structure, Zeitler *et al.* Eur J Neurosci, 2016) that Diazepam acts within 15-30 min then is relayed by neurosteroidogenesis. Therefore, it is difficult to conclude on the primary effect alone.

I have read this submission. I believe that I have an appropriate level of expertise to confirm that it is of an acceptable scientific standard.

# Response

**Published:** 2017-06-14

**Authors:** Stephen Humble

**Affiliations:** Imperial College London, UK

Dear Prof Darbon,

Thank you for your comments and for taking the time to review the paper. I agree that the illustration and description could be improved and I will do so as per your suggestions. I agree that the brief background description makes the research harder for a broad readership- this is as a result of the article’s word limits.

Regarding the 2 hour incubation period for diazepam. This issue was considered at length during the experimental design stage and this is a relevant concern. It is of course true that *in vivo* diazepam acts within 15-30 minutes and that *in vivo* propofol and etomidate may act within seconds when given intravenously. However, previous work (Benkwitz *et al.,* 2007; Gredell *et al.,* 2004) has shown that in brain slice preparations the duration of onset may be much longer in lipophilic compounds such as these because the drug has to diffuse through the slice to reach the GABA _A_ receptor on the neurone located within the slice. This contrasts to rapid drug delivery via an intact *in vivo *circulation, which may deliver the drug directly to the relevant neurone within seconds to minutes. Taking all this into account it was decided to use a 2 hour incubation period in order to maximise the impact of diazepam and highlight the difference between the diabetic neurones and the wild type. i.e. that diazepam in the presence of flumazenil (benzodiazepine receptor antagonist) had a greater response in the diabetic neurones, which appears to be mediated via upregulated neurosteroidogenesis and could be blocked by finasteride. Finally, I think that these results are not inconsistent with the Zeitler *et al.*, 2016 paper.

Yours sincerely

Stephen Humble

# Peer review: Referee response for version 1

**DOI:** 10.5256/f1000research.11924.r22166

**Published:** 2017-05-30

**Authors:** Slobodan M. Todorovic

**Affiliations:** 1 Department of Anesthesiology, School of Medicine, University of Colorado Anschutz Medical Campus, Aurora, CO, USA

My major concern with this study is that title and discussion are not relevant to the data presented. There is no justification to link this study to neuropathic pain or painful diabetic neuropathy. Perhaps more appropriate title would be:

 “Mitochondrial dysfunction in an animal model of Type 2 diabetes  may be involved reduction in neurosteroid synthesis “ 

I have read this submission. I believe that I have an appropriate level of expertise to confirm that it is of an acceptable scientific standard, however I have significant reservations, as outlined above.

# Response

**Published:** 2017-05-31

**Authors:** Stephen Humble

**Affiliations:** Imperial College London, UK

Dear Prof Todorovic,

Thank you for taking the time to review the paper. I agree that the title should be changed. I have reworded it as follows in relation to your comments:

“Mitochondrial dysfunction in an animal model of diabetic neuropathy is associated with a reduction of neurosteroid synthesis”

With the benefit of hindsight I realize that linking these data in isolation directly to neuropathic pain and painful diabetic neuropathy is not justified. For the purposes of brevity full rationale was not explained in as much depth as was required to explain the link fully. If this paper is viewed alongside this other recent paper then the scientific rationale and justification may be understood:

Humble SR: Neurosteroids are reduced in diabetic neuropathy and may be associated with the development of neuropathic pain [version 1; referees: 1 approved, 2 approved with reservations]. F1000Res. 2016a; 5: 1923.

I will make some modifications to the abstract and discussion in relation to your comments as follows:

**Abstract section**

Old (final sentence):

In diabetic neuropathic pain, mitochondrial dysfunction may play an important role. Enhancing the GABAergic neurosteroid tone could be of potential therapeutic benefit.

New:

In diabetic neuropathy, mitochondrial dysfunction may play an important role. Enhancing the GABAergic neurosteroid tone could be of potential therapeutic benefit.

**Discussion section**

Old (final sentence):

With reference to diabetic neuropathy, mitochondrial dysfunction may play an important role, and enhancing the GABAergic neurosteroid tone directly or indirectly could be of potential therapeutic benefit.

New:

Considering the findings in this paper alongside previous work (Humble, 2016a) it appears that mitochondrial dysfunction may play an important role in the development of type 2 diabetic neuropathy. In this context, it follows that enhancing the GABAergic neurosteroid tone directly or indirectly could be of potential therapeutic benefit for diabetic neuropathic pain and hypersensitivity.

Yours sincerely

Stephen Humble
